# Impact of tumor chronology and tumor biology on lymph node metastasis in breast cancer

**DOI:** 10.1186/2193-1801-2-480

**Published:** 2013-09-23

**Authors:** Ann Smeets, Andries Ryckx, Ann Belmans, Hans Wildiers, Patrick Neven, Giuseppe Floris, Patrick Schöffski, Marie-Rose Christiaens

**Affiliations:** Multidisciplinary Breast Center, KU Leuven, University Hospitals Leuven, Leuven, Belgium; Department of Public Health and Primary Care, KU Leuven, Interuniversity Institute for Biostatistics and Statistical Bioinformatics, Leuven, Belgium; Department of Oncology, KU Leuven, Surgical Oncology, University Hospitals Leuven, Leuven, Belgium; Department of Oncology, KU Leuven and Department of General Medical Oncology, Leuven Cancer Institute, University Hospitals Leuven, Laboratory of Experimental Oncology (LEO), Leuven, Belgium; Department of Oncology, KU Leuven, Gynaecological Oncology, University Hospitals Leuven, Leuven, Belgium; Department of Imaging and Pathology, KU Leuven, University Hospitals Leuven, Leuven, Belgium

**Keywords:** Tumor chronology, Tumor biology, Lymph node, Metastasis, Breast cancer

## Abstract

**Synopsis:**

The significance of nodal metastasis in breast cancer is under discussion. We investigated the impact of variables of tumor chronology and tumor biology on the presence of lymph node metastases.

**Purpose:**

Lymph node involvement is the main prognostic factor in breast cancer. However, it is under discussion whether nodal metastasis in breast cancer only reflects the chronological age of the tumor or whether it is also a marker of tumor biology. The goal of our study was to investigate the impact of variables of tumor chronology and biology on the presence of lymph node metastases.

**Methods:**

We performed a retrospective analysis of data from 3002 patients with an early invasive breast carcinoma. All patients underwent primary surgery at the University Hospitals Leuven between 2001 and 2009. First, the impact of tumor size on the presence of lymph node metastasis was evaluated as the chronological age of a tumor is supposed to be reflected in its size. Next, the impact of tumor grade, lymphovascular invasion and the hormone receptor status, which are all variables of tumor biology, was studied. Logistic regression analyses were performed and the area under the ROC curve (AUC) was calculated as a measure of discrimination between logistic regression models.

**Results:**

Using pathological tumor size the AUC of prediction was 0.67. Based on variables of tumor biology, axillary lymph node positivity could be predicted with an AUC of 0.68. Combining variables of tumor chronology and biology an AUC of 0.74 for the prediction of axillary lymph node (ALN) positivity was calculated.

**Conclusions:**

According to our data variables of tumor chronology and tumor biology have a similar impact on the presence of lymph node metastasis.

## Introduction

Lymph node involvement is the main prognostic factor in breast cancer and a crucial component of the staging system. However, the significance of nodal metastasis in breast cancer is under discussion. It has been considered a reflection of the chronological age of the tumor since the time of Halsted (Mittra 1993; Tubiana-Hulin et al. 1995; Paterson et al. 1982; Mittra & MacRae 1991). This view was based on the concept that breast cancer always spreads to the regional lymph nodes first and to distant sites afterwards. The improved prognosis of patients with node-negative tumors was attributed to timely resection of the tumor. An alternative reason for the difference in prognosis between node-negative and node-positive patients could be that the nodal status in breast cancer is also a marker of tumor biology (Jatoi et al. 1999). Understanding the significance of lymph node involvement could provide important insights into cancer growth and allow more rational decision-making in clinical practice.

The primary goal of this study was to evaluate the impact of variables of tumor chronology and those of tumor biology on lymph node positivity. The chronological age of the tumor is supposed to be reflected through its size. It is well established that there is a strong relationship between the pathological size of a tumor (pT) and axillary lymph node metastasis (Carter et al. 1989).Variables of tumor biology are tumor grade, lymphovascular invasion (LVI), the hormone receptor status (ER/PR/HER-2) and multifocality. The correlation of these variables with the presence of lymph node involvement has been investigated extensively (Patani et al. 2007). In our study, multifocality was not studied as we included only unifocal tumors.

Secondly, we assessed whether accurate prediction of lymph node involvement is possible preoperatively, as in the preoperative setting only clinical tumor size (cT), tumor grade and the hormone receptor status are known.

## Patients and methods

### Patients

Data were obtained by retrospective review of the Multidisciplinary Breast Center database from the University Hospitals Leuven (Leuven, Belgium). Patients with a primary operable breast cancer between 1 January 2001 and 31 December 2009 were included in the study. The local surgical treatment consisted of wide excision of the tumor followed by radiotherapy, or mastectomy with or without radiotherapy. All patients underwent axillary staging by sentinel lymph node (SLN) biopsy and/or axillary lymph node dissection. We excluded patients (i) treated for a local recurrence, (ii) with only a carcinoma in situ, (iii) who received neo-adjuvant therapy, (iv) with primary metastatic disease and (v) with a multifocal tumor.

Tumor size and nodal status were defined according to TNM standards (AJCC) (Greene 2002). cT is the largest measured tumor size using clinical examination and imaging (mammography, ultrasound or MRI). pT is the largest diameter measured on final pathology of representative slides of the surgical resection specimen. Pathological node positivity included micro- and macrometastases.

### Sentinel lymph node localization

The SLN procedure was performed by injection of a radioactive (^99m^Tc-labelled nanocolloid) tracer at the level of the tumor and Patent Blue V dye® (Guerbet, France) retroareolar. The SLNs were removed surgically with the assistance of a hand-held gamma-ray detection probe.

### Examination of the lymph nodes

SLNs were routinely examined by serial sectioning. Every 300 μm 2 sections were stained, 1 with routine haematoxylin and eosin (H&E) and 1 stained immunohistochemically using monoclonal antibodies against cytokeratin. Lymph nodes in an axillary lymph node dissection were examined by H&E staining using 3 sections per node. According to published guidelines, lymph nodes from lobular breast cancers, classified as lymph node negative on H&E, were additionally stained with epithelial markers (Greene 2002).

### Statistical methodology

Clinical characteristics were summarized with absolute number (n), mean and standard deviation for continuous variables and by observed counts for categorical variables. The correlation between the selected variables of tumor chronology and biology and lymph node involvement was determined by using logistic regression analysis. Only patients with all available data were included. The area under the ROC curves (AUC) were calculated to discriminate between both regression models. The difference between the two models was assessed using the methods described by DeLong, DeLong and Clarke-Pearson (DeLong et al. 1988). The impact of variables of tumor biology on the presence of lymph node metastasis was measured by the combination of tumor grade, LVI, ER, PR and HER-2.

The AUC of a model including all variables known in the preoperative setting, was calculated and compared with the AUC of the model including all variables known in the postoperative setting.

All analyses were performed using SAS software, version 9.2 (SAS Institute, Cary, NC).

## Results

Out of a total database of 10390 patients, 4124 patients were found to meet the inclusion criteria. Patients with missing tumor data were excluded, resulting in 3002 tumors for analysis. The mean age was 57.8 years. The mean pathological tumor size was 22.2 mm. A sentinel lymph node biopsy was performed in 1476 patients, followed by an axillary lymph node dissection only when the sentinel lymph node was positive. The remaining 1526 patients underwent an axillary lymph node dissection. Fifty-nine percent of patients were node-negative and 41% were node-positive. Thirty percent of patients were pN1, 7% pN2 and 4% pN3

### Univariate analysis

Table 1 shows the univariate analysis between clinicopathological characteristics and the presence of lymph node metastasis. Positive nodal status was significantly correlated with cT, pT, tumor grade, the presence of LVI and HER-2. No significant correlation was found between lymph node involvement and ER and PR.

**Table 1:** **Univariate analysis between clinicopathologial characteristics and the presence of lymph node metastasis**

Patient characteristic		Negative		Positive		Total		P-value
		N = 1778		N = 1224		N = 3002		
**Age (**mean ± SD)		57.8 ± 12.42		57.5 ± 12.48		57.7 ± 12.45		
**Tumor size** (mean ± SD)		22.2 ± 13.83		35.6 ± 24.33		27.7 ± 19.94		
**cT** (n,%)	1	1015	(57.09)	430	(35.13)	1445	(48.13)	<.001
	2	683	(38.41)	636	(51.96)	1319	(43.94)	
	3	71	(3.99)	127	(10.38)	198	(6.60)	
	4	9	(0.51)	31	(2.53)	40	(1.33)	
**pN** (n,%)	0	1778	(100)	0	(0)	1778	(59.23)	<.001
	1	0	(0)	907	(74.10)	907	(30.21)	
	2	0	(0)	211	(17.24)	211	(7.03)	
	3	0	(0)	106	(8.66)	106	(3.53)	
**Tumor grade** (n,%)	1	317	(17.83)	130	(10.62)	447	(14.89)	<.001
	2	761	(42.80)	519	(42.40)	1280	(42.64)	
	3	700	(39.37)	575	(46.98)	1275	(42.47)	
**LVI** (n,%)	No	1606	(90.33)	709	(57.92)	2315	(77.12)	<.001
	Yes	172	(9.67)	515	(42.08)	687	(22.88)	
**ER** (n,%)	Negative	275	(15.52)	186	(15.20)	462	(15.39)	0.807
	Positive	1502	(84.48)	1038	(84.80)	2540	(84.61)	
**PR** (n,%)	Negative	465	(26.15)	322	(26.31)	787	(26.22)	0.925
	Positive	1313	(73.85)	902	(73.69)	2215	(73.78)	
**HER-2** (n,%)	Negative	1608	(90.44)	1064	(86.93)	2672	(89.01)	0.003
	Positive	170	(9.56)	160	(13.07)	330	(10.99)	

### Logistic regression analysis

#### Impact of variables of tumor chronology on pN

As mentioned before, the chronological age of the tumor is supposed to be reflected through its size. In the preoperative setting only the cT is known, in the postoperative setting the pT is used. Logistic regression analysis showed that in a model only including chronological tumor characteristics, the presence of lymph node metastases could be predicted with an AUC of 0.62 when cT was used and with an AUC of 0.67 when pT was used (Table 2).

**Table 2:** **Logistic regression analyses for the prediction of the presence of lymph node metastasis by variables of tumor chronology and biology**

Fitted model (N = 3002)	Predictor	Effect	Odds ratio (95% CI)	P-value	AUC (95% CI)
**cT**	cT	1 vs 4	0.123 (0.058; 0.2610)	<.0001	0.6231 (0.6048; 0.6414)
		2 vs 4	0.270 (0.128; 0.572)		
		3 vs 4	0.519 (0.234; 1.152)		
**pT**	pT	1 vs 4	0.129 (0.050; 0.332)	<.0001	0.6700 (0.6522; 0.6877)
		2 vs 4	40.352 (0.137; 0.906)		
		3 vs 4	1.338 (0.501; 3.575)		
**LVI**	LVI	Yes vs No	6.782 (5.587; 8.234)	<.0001	0.6620 (0.6466; 0.6775)
**Grade**	Grade	1 vs 3	0.499 (0.396; 0.630)	<.0001	0.5531 (0.5341; 0.5721)
		2 vs 3	0.830 (0.710; 0.971)		
**ER**	ER	Neg vs Pos	0.975 (0.797; 1.194)	0.8076	0.5016 (0.4885; 0.5148)
**PR**	PR	Neg vs Pos	1.008 (0.854; 1.189)	0.9248	0.5008 (0.4848; 0.5168)
**HER–2**	HER-2	Neg vs Pos	0.703 (0.559; 0.884)	0.0026	0.5176 (0.5059; 0.5292)
**grade, ER, PR, HER–2**					0.5649 (0.5448; 0.5850)
**cT, grade, ER, PR, HER–2**					0.6406 (0.6206; 0.6606)
**LVI, grade, ER, PR, HER–2**					0.6852 (0.6657; 0.7047)
**pT, LVI, grade, ER, PR, HER–2**					0.7462 (0.7279; 0.7645)

#### Impact of variables of tumor biology on pN

Next, logistic regression analyses of models including variables of tumor biology were performed. A model including LVI could predict lymph node positivity with an AUC of 0.66. Models including tumor grade, ER, PR or HER-2 could only predict lymph node involvement with AUCs of 0.55, 0.50, 0.50 and 0.51 respectively. Discrimination accuracy for lymph node involvement of tumor grade combined with LVI, ER, PR and HER-2 was 0.68 (Table 2).

#### Impact of variables of tumor chronology plus tumor biology on pN

When combining pT and variables of tumor biology axillary nodal positivity could be predicted with an AUC of 0.74. In the preoperative setting, the presence of lymph node metastasis could be predicted with an AUC of 0.64 (Table 2).

#### Comparison of ROC curves

**Figure 1:**
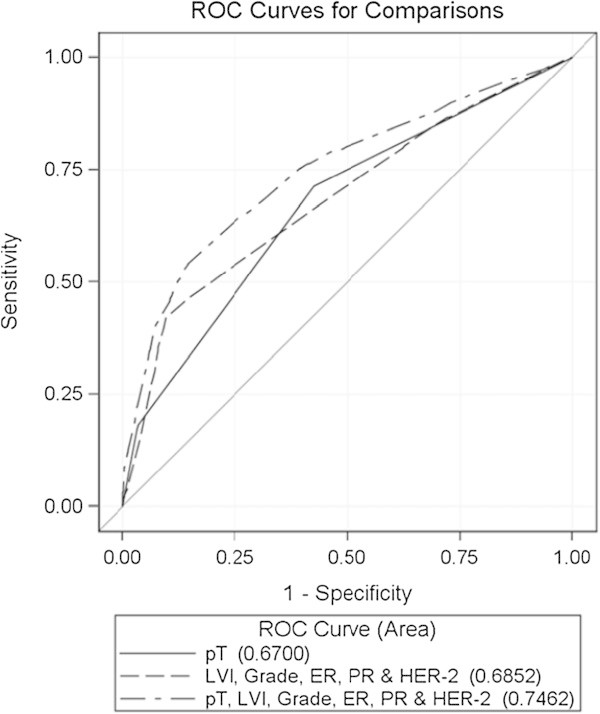
**ROC curves for the prediction of the presence of lymph node metastasis by variables of tumor chronology (pT), tumor biology (LVI, grade, ER, PR & HER-2) and the combination of both.**

**Figure 2:**
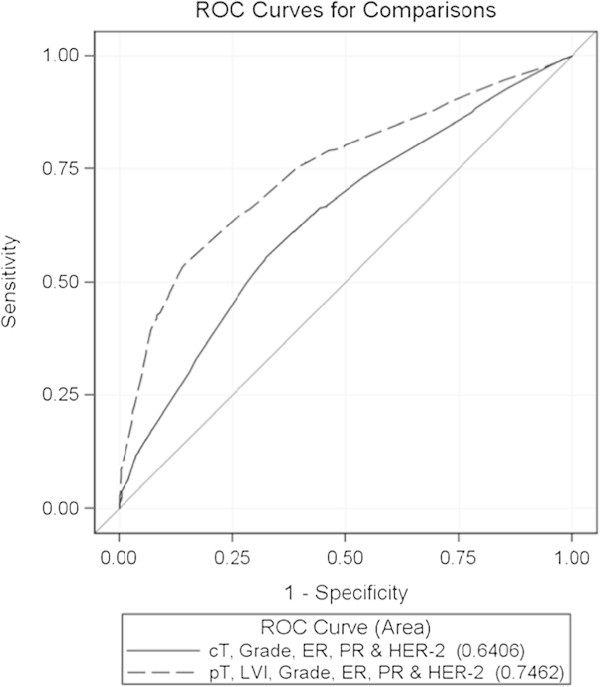
**ROC curves for the prediction of the presence of lymph node metastasis by variables known in the preoperative (cT, grade, ER, PR & HER-2) and the postoperative (pT, LVI, grade, ER, PR & HER-2) setting.**

Comparison of ROC curves showed that (i) the impact of variables of tumor chronology and tumor biology on lymph node positivity was similar, (ii) the addition of variables of tumor biology to variables of tumor chronology significantly improved the prediction of lymph node metastasis (p<0.0001) and (iii) prediction of the presence of lymph node metastasis in the postoperative setting was significantly better than in the preoperative setting (p<0.0001) (Figure 1 and Figure 2).

#### Missing data

The exclusion of patients with missing data could introduce serious bias into the results, unless the missing data is completely at random (MCAR scenario). The bias by excluding patients without available data was probably small in this study because the odds ratios from univariate analysis were similar when all patients were included as when only patients with all available data were included (data not shown).

## Discussion

The results of this study show that lymph node involvement in breast cancer reflects both tumor chronology and biology. Using pathological tumor size, lymph node involvement can be predicted with an AUC of 0.67. The combination of variables of tumor biology can predict lymph node involvement with an AUC of 0.68. Thus, based on our data, tumor chronology and biology seem to have a similar impact on the lymph node status. When using the combination of pathological tumor size, LVI, tumor grade, ER, PR and HER-2 the lymph node status can be predicted with an AUC of 0.74.

The chronological age of a tumor can be considered as a product of the tumor size and host resistance to the tumor divided by its biological aggressiveness (Mittra 1993). In this study, we used only tumor size to measure the chronological age of the tumor as it is evident that tumor size increases over time and is easily measurable. On the other hand, host resistance to the tumor and the biological aggressiveness of the disease are less accurately defined. The strong direct relationship between the size of a tumor and axillary lymph node metastasis is well established (Fisher et al. 1969). An analysis of data on 24740 cases of breast cancer recorded in the SEER program of the National Cancer Institute has demonstrated that this relationship is strictly linear (Carter et al. 1989).

Besides tumor size, variables of tumor biology have been shown to correlate with axillary lymph node involvement (Patani et al. 2007; Yoshihara et al. 2012). We used LVI, tumor grade, ER, PR and HER-2 to evaluate the impact of tumor biology on the presence of lymph node metastasis. ER, PR and HER-2 receptor status have not been found to be consistently related to lymph node status. However, we included them in the analysis as they are important variables of tumor biology. Multifocality was not evaluated as only unifocal tumors were included in our study. The addition of tumor grade, ER, PR and HER-2 to LVI had only a limited impact on the prediction of the lymph node status (AUC 0.66 => 0.68).

It could be questioned whether LVI reflects tumor biology or tumor chronology. Rakha et al. recently demonstrated that it is an independent prognostic factor. **U**nivariate analysis showed a significant correlation between LVI and tumor size and between LVI and the nodal status but in multivariate analysis LVI remained an independent predictor of survival. Multivariate analysis including the interaction between LVI and lymph node status and tumor size, did not result in significant P_interaction_ values, indicating that the effects of LVI on patient outcomes were not affected significantly by the lymph node status or tumor size (Rakha et al. 2012). Moreover, LVI can not simply be considered as the first step in the process of lymph node involvement as in our series 57% of the tumors from patients with lymph node positive breast cancer did not show LVI. These data confirm that LVI is a parameter of tumor biology.

To evaluate whether the lymph node status can be accurately predicted in the preoperative setting, we determined the impact of cT, tumor grade, ER, PR and HER-2 on the lymph node status. Based on these variables, axillary lymph node positivity could be predicted with an AUC of 0.64. There is no perfect correlation between the tumor grade on core biopsy and on the resection specimen. Hence, it might be that the actual AUC is even lower than 0.64. These data demonstrate that an accurate prediction of the lymph node status is not possible in the preoperative setting.

Mittra et al. did not find a significant correlation between most biological prognostic factors and lymph node involvement and thus concluded that the axillary node status is merely a reflection of the chronological age of breast cancer. However, their data showed a significant correlation between tumor grade and the lymph node status. So, their conclusion does not seem to be fully supported by their data. Moreover, they did not include LVI as a biological prognostic factor while, as mentioned above, LVI has been shown to be an independent prognostic factor (Rakha et al. 2012).

Our findings are in line with these of Jatoi et al. (Jatoi et al. 1999). They showed that patients with four or more involved nodes at initial diagnosis have a significantly worse outcome after relapse than patients with node-negative breast cancer, and thus that nodal metastasis is not only a marker of diagnosis at a later point in the evolution of the disease but also a marker of an aggressive phenotype.

Understanding the significance of lymph node involvement provides insight into cancer growth and control that can be applied in clinical practice. For example, based on our data, tumor size should not determine the selection of patients for a sentinel lymph node procedure as (1) variables of tumor chronology and tumor biology have a similar impact on the lymph node status and (2) prediction of the lymph node status based on clinical tumor size is not accurate (AUC 0.62). Moreover, the findings of this study allow for more individualized clinical decision making, for example to estimate the risk of lymph node involvement in patients with an unexpected finding of an invasive tumor on final pathology.

Several nomograms have been developed to predict the likelihood of sentinel lymph node metastases, based on clinical variables. A nomogram developed at the Memorial Sloan-Kettering Cancer Center (New York, NY) includes nine variables: age, tumor size, tumor type, LVI, multifocality, nuclear grade, tumor location, and ER and PR status (Bevilacqua et al. 2007). It can predict the presence of lymph node metastases in the sentinel lymph node with an AUC of 0.75. This is a reasonable prediction rate.

By combining variables of tumor chronology and biology, we were able to predict ALN positivity with an AUC of 0.74. Many clinically useful predictive models have an AUC in this range. (van la Parra et al. 2013)

Nevertheless, an AUC of 0.74 indicates that, besides the chronological age of the tumor and specific biological features, other variables have an impact on the lymph node status. One such variable is the location of the tumor in the breast. Patients with lateral and retroareolar tumors have a higher probability of positive lymph nodes compared to patients with medial tumors (Yoshihara et al. 2013). Moreover, the lymph node status might be a marker of the host response to the tumor. Hence, it could be hypothesized that a weakened host response results in early metastasis to the axillary lymph nodes (Jatoi et al. 1999).

## Conclusion

According to our data, variables of tumor chronology and tumor biology seem to have a similar impact on the presence of lymph node metastasis. Based on pathological tumor size, the presence of LVI, tumor grade, ER, PR and HER-2, the presence of axillary lymph node metastasis can be predicted with an AUC of 0.74.

### Ethical standards

No experiments were performed.
